# Development of High-Performance Supercapacitor based on a Novel Controllable Green Synthesis for 3D Nitrogen Doped Graphene

**DOI:** 10.1038/s41598-018-37369-x

**Published:** 2019-02-04

**Authors:** Noha A. Elessawy, J. El Nady, W. Wazeer, A. B. Kashyout

**Affiliations:** 1 Advanced Technology and New Materials Research Institute, City of Scientific Research and Technological Applications (SRTA-City), New Borg El-Arab City, P.O. Box 21934, Alexandria, Egypt; 2 Electronic Materials Department, Advanced Technology and New Materials Research Institute, City of Scientific Research and Technological Applications (SRTA-City), New Borg El-Arab City, P.O. Box 21934, Alexandria, Egypt

## Abstract

3D sponge nitrogen doped graphene (NG) was prepared economically from waste polyethylene-terephthalate (PET) bottles mixed with urea at different temperatures using green approach via a novel one-step method. The effect of temperature and the amount of urea on the formation of NG was investigated. Cyclic voltammetry and impedance spectroscopy measurements, revealed that nitrogen fixation, which affects the structure and morphology of prepared materials improve the charge propagation and ion diffusion. The prepared materials show outstanding performance as a supercapacitor electrode material, with the specific capacitance going up to 405 F g^−1^ at 1 A g^−1^. An energy density of 68.1 W h kg^−1^ and a high maximum power density of 558.5 W kg^−1^ in 6 M KOH electrolytes were recorded for the optimum sample. The NG samples showed an appropriate cyclic stability with capacitance retention of 87.7% after 5000 cycles at 4 A g^−1^ with high charge/discharge duration. Thus, the prepared NG herein is considered to be promising, cheap material used in energy storage applications and the method used is cost-effective and environmentally friendly method for mass production of NG in addition to opening up opportunities to process waste materials for a wide range of applications.

## Introduction

Electrochemical capacitors (ECs) are considered as one of the most promising energy storage technologies responding to the increasing demand of the clean energy related technologies and applications such as portable/mobile electronics, electrical vehicles, or other storage systems based on sources like windmills and solar cells.

The graphene-based materials are promising for applications in supercapacitors and other energy storage devices and there are many researches on the refinement of their structures and development of effective low cost synthesis techniques as well as possible clarification of their electrochemical performance^1^.

Chemical doping has been exceedingly used to enhance capacitor property of graphene. Doping of graphene with nitrogen is of much interest, since it improves the overall conductivity and supercapacitive properties because introducing N atoms intrinsically improve the electronic properties of graphene in particular the performance as supercapacitor electrodes^2–4^. Among a variety of nitrogen sources, urea has a high nitrogen content (46 wt %), and can react with oxygen-containing groups. Hence, urea is a typical precursor that has been used widely as a nitrogen source in experiments on graphene^3,5–7^.

There are numerous methods to synthesize nitrogen doped graphene, which can be categorized into two kinds of methods: direct synthesis such as chemical vapor deposition (CVD), arc-discharge, segregation growth, solvothermal methods, and post treatment such as hydrazine hydrate, thermal and plasma treatments^5,8^. However, the extremely low yield and high cost of these methods limit their application only to fundamental studies. Later, it was found out that GO can be used as a platform for N incorporation via thermal annealing with NH_3_ or nitrogen plasma treatment but these methods involve either toxic precursors or need sophisticated equipment^5^. Many researchers aim to develop one-step simple preparation way for inquiring nitrogen doping into graphene and most of their efforts have used aqueous mixture of graphene oxide (GO) and nitrogen source as starting the materials. For instance, Hao *et al*. prepared novel cross-linking three-dimensional (3D) graphene layers utilizing gas-foaming technology by using colloidal solution of exfoliated GO and NH_4_Cl solution; the obtained material showed a capacitance of 231.2 F g^−1^ at a current density of 1 A g^−1^ in 1 M H_2_SO_4_^9^. Chen *et al*. produced 3D graphitic carbon nitride functionalized graphene networks by the hydrothermal reduction of GO sheets and exfoliated carbonized melamine in their mixed aqueous dispersions at 180 °C for 12 h. The obtained material displayed 264 F g^−1^ at a current density of 0.4 A g^−1^ in 0.1 M LiClO_4_^10^. Xiao *et al*. inserted nitrogen into graphene using nitric acid through thermal treatment at 500 °C, which showed a capacitance value of 370 F g^−1^ at 1 A g^−1^ in 6 M NaOH^11^. Zhang *et al*. produced nitrogen doped graphene using nitric acid and a high temperature co-processing method at 500 °C for 4 h in a quartz tube furnace with a high specific surface area (436.7 m^2^ g^−1^) and a high specific capacitance (481 F g^−1^, 1 A g^−1^)^12^.

Therefore, developing a low-cost, scalable, and eco-friendly method is still of great interest. Herein, the nitrogen (N) doping was used to improve the capacitance performance of graphene through a novel synthesis using PET waste bottles and urea. Urea has two amine groups with nitrogen content about 46 wt% joined by a carbonyl (C=O) functional group. Therefore, it is a good candidate to be used as nitrogen precursor. Moreover, the presence of H^+^ ions induce the hydrogenation of dangling bonds on the formed graphene layer^13,14^, and hence, forming a strong C–H bond. Bonding configuration changes from sp^2^ to sp^3^ and less oxygen functional groups are present in grapheme, that in turn enhances the capacitive behavior because the oxygen functional groups would impede the migration of electrolyte ions into micropores and as a result reduced the effective pore volume of the graphenic materials. Both raw materials are low cost and are manufactured in large quantities. The great challenge is the conversion of waste PET bottles using a one step thermal decomposition method into supercapacitor electrode material through simple and cost effective method. and that was demonstrated by using only urea. In this method, graphene gets simultaneously doped with nitrogen.

## Results and Discussions

Thermogravimetric analysis (TGA) is used to investigate the behaviors of PET and urea in the thermal decomposition process. Supplementary Fig. S1 presents the thermogravimetric curves of urea, PET/urea (a mixture of PET and urea with 1:1 weight ratio) and PET. The curve of urea indicates three stages of mass loss. In the first stage which is related to urea decomposition to biuret and volatile products whereas a mass loss about 79% occurs between 150 to 240 °C. The second stage up to 300 °C involves a distinct mass loss of 20%, which corresponds to the generation of cyanuric acid and volatile materials. Moreover, small amounts of ammelide, ammeline and melamine are formed. These small amounts are decomposed during the last step up to 400 °C^6^. Urea totally decomposes into volatile products with a total weight loss close to 600 °C. While, for PET, the degradation started at 360 °C and achieve maximum degradation rate at 480 °C. The curve exhibits one major mass loss which is attributed to thermal decomposition and the residue at 800 °C is 0.9 wt%. This is obviously associated with the segment corresponding to the carboxylic acid and the benzene ring appearing in the chemical unit of PET^15^. Compared to PET and urea curves, the curve of PET/urea is coalesce of their two curves. A mass loss of 60% took place in the first step at the temperature range of 150–240 °C, which is in good agreement with urea decomposition to biuret. This process was followed by two steps which ended at 480 °C similar to PET curve. During the thermal decomposition process, the out-gassing as CO, CO_2_, NO_2_, and H_2_O molecules could introduce force to expand the graphene layers and help the formation of porous frameworks^11,16^. That is confirmed by SEM and TEM images. Meanwhile, the embedded nitrogen atoms in the carbon lattice cause the formation of NG frameworks. In order to prove the presence of nitrogen doped in graphene structure, an elemental analysis was conducted on the dried product with XPS and FTIR. The mass fractions of carbon, oxygen, and nitrogen for all samples are presented in Supplementary Table S2.

**Figure 1:**
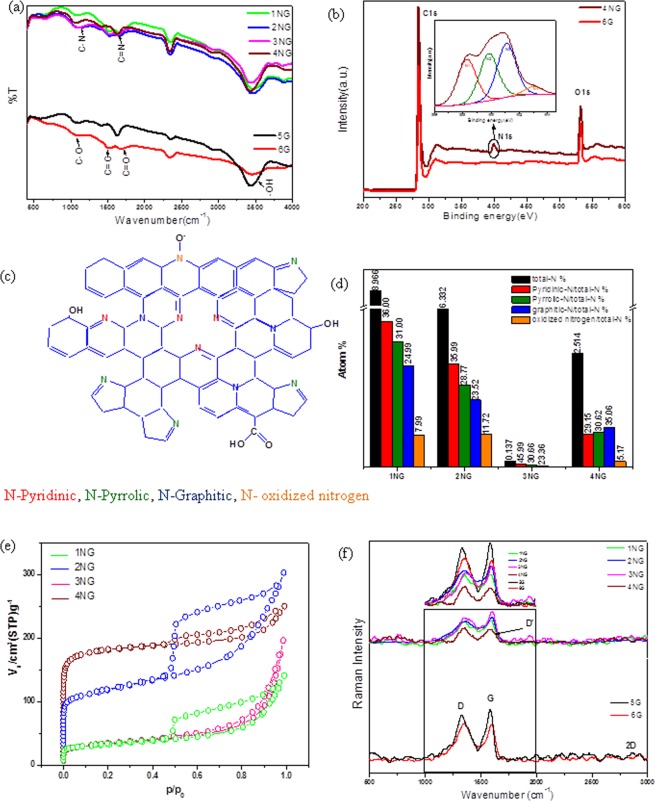
Materials characterization. (**a**) FTIR, (**b**) XPS spectra survey (inset) high resolution N1s XPS spectra of 4NG sample, (**c**) Schematic suggested structure of NG based on the result of XPS analysis, (**d**) atomic % of nitrogen in every NG sample and their components, (**e**) Nitrogen adsorption-desorption isotherms of nitrogen doped graphene samples and and (**f**) Raman spectra.

As shown in Fig. 1(a) Fourier Transform Infrared (FT-IR) spectroscopy was used to investigate the chemical bonding compositions and functional groups of graphene and NG prepared samples. All samples exhibit a distinct broad absorption at ∼3445 cm^−1^ corresponding to the stretching vibration mode of -OH and a characteristic peaks appearing at 1532 cm^−1^ correspond to the skeletal vibration of C=C (sp^2^ hybridization)^10^. It is noticeable from the spectrum of undoped graphene samples (5 G and 6 G) that the intensities of characteristic peaks decreased by rising the temperature of PET thermal decomposition and this indicates that the quality of produced graphene increases with increasing the treatment temperature due to decreasing amount of oxygenous groups. However, for NG samples, the spectra show marked changes in the 1000–1700 cm^−1^ region, particularly in the C–O stretching bands, in addition to the C=N stretching vibration modes which appeared at 1631 cm^−1^ and that confirm the success introducing of N atoms into graphene lattice. Complementary information on nitrogen functional groups were obtained by X-ray photoelectron spectroscopy (XPS) analysis as shown in Fig. 1(b), where N atoms eventually appear when doped into graphene in different configurations, such as graphitic-N, pyridinic-N and pyrrolic-N^16^. The XPS spectra of the graphene sample derived from PET bottles waste at 800 °C (sample 6 G) confirmed that carbon and oxygen were the only present atoms and nitrogen signal could be clearly observed in the XPS spectra of NG samples. For NG samples (Supplementary Fig. S2(a)), the high-resolution C1s peak corresponding to sp^2^-hybridization (C-C bond, 284.7 eV) was observed and this demonstrate that C atoms were arranged in a honeycomb lattice and the fitted peaks centered at 285.55, 286.7, 287.85, and 288.95 eV are assigned to C=N, C-N, C=O, and O=C-O, respectively. It is worth noting that a small amount of oxygen containing functional groups was in samples because the presence of oxygen functional groups on the surface of graphenic materials reduced its surface conductivity and prevented ions from entering into the pore^17^. But they have a great influence on the capacitive performance^18^ because it improves the hydrophilicity of the surface which enhancing the ions diffusion of the aqueous electrolyte and reduced the mass transfer resistance on the surface of electrode; subsequently make it easier for ions to form electrochemical double layer and as a result the capacitance will be increased^17,18^.

However, the high-resolution N1s spectra of the N-doped graphene in Fig. 1(b) can also be decomposed into main four different peaks that can be assigned to pyridinic N (~398.55 eV), pyrrolic N (~400.6 eV), graphitic N (~401.95 eV) and oxidized N atoms (∼403 eV). The results suggest that the N atoms are covalently bonded networks of graphene and there are two possible ways; the first suggested way is via chemical reactions of the urea with surface functional groups in the intermediate carbon structure formed during decomposition of PET and subsequent thermal transformations of the formed compound^1^. The second way for N-doping is that carbon nitride, produced by the decomposition of the N precursors, acts as an intermediate for the formation of N dopant in the NG. This way had a low handout here because the yield of carbon nitride from urea was very low and FTIR spectroscopy had confirmed the absence of the signature peak for carbon nitride at ~810 cm^−1^ indicating that it is unlikely that carbon nitride is involved in this process. Moreover, it was notable from XPS high-resolution N1s spectra for sample 1NG and 4NG (Supplementary Fig. S2(b,c)) that the reaction temperature was found to play a key role in the distribution of N atoms into the graphene structure. For instance, the oxidized nitrogen functionalities at 403 eV disappeared, pyridinic species decreased and pyrrolic species increased as the decomposition temperature increased from 600 °C to 800 °C. Furthermore, it was observed that the graphitic -N peak at 401.1 eV (Supplementary Fig. S2(c)), became increasingly obvious at a higher decomposition temperature. The favorable formation of graphitic -N at high temperatures can be attributed to its thermal stability, higher than that of pyrrolic-N and pyridinic-N^6,19^. This suggests that the mutual transformation among nitrogen functionalities is rather easy at such a temperature range. Meanwhile, by increasing the decomposition temperature, the nitrogen content decreased from 8.969 at.% (sample 1NG) to 2.514 at.% (sample 4NG).

As shown in Fig. 1(d), according to the results of XPS, with the decreasing of the urea amount the nitrogen content also decreased from 8.969 at.% to 0.137 at.% for samples obtained at the same decomposition temperature, which means that urea is of significant importance for nitrogen doping. These results give evidence of the effect of the thermal decomposition temperature and PET to urea ratio on formed nitrogen functionalities ratio.

Nitrogen adsorption–desorption isotherm curves for NG samples as shown in Fig. 1(e) were elucidated as type-IV isotherm according to IUPAC classification^8,13^ with distinct hysteresis loops at the range of relative pressures (P/P_0_) from 0.45 to 1.0 which can result from the coexistence of micropores and mesopores. BET surface areas of all samples are illustrated in Supplementary Table S3 which are calculated from the desorption branch of isotherm. According to the BET surface areas and by comparing NG with undoped graphene, which was prepared at the same temperature, it was noted that the surface area increased with doping and that can be attributed to 3D porous structure of N-doped samples. Meanwhile, for the sample treated at 600 °C as nitrogen content was further increased, the surface area decreased and that can be attributed to partial agglomeration which restricts the surface area increase^20,21^. The pore size distributions of N doped samples are shown in Supplementary Information Fig. S4. All samples contain micropores less than 2 nm in size, in addition to many mesopores and macropores are not present. However, it is generally accepted that a high surface area can provide more active sites for charge exchange to increase specific capacitance, and the mesoporous structure can improve the electrolyte infiltration and facilitate the ion diffusion^13^.

XRD patterns, illustrated in Supplementary Fig. S3, show a broad peak at 23.8° which corresponds to (002) peak of graphene crystal plane. The diffraction peak of NGs located at 25.5° and the position of the (002) peak was slightly left-shifted compared to graphene, confirming the change of the structure. It is interesting to note that by increasing the thermal decomposition temperature and nitrogen doping, the broadening of the peaks decreases due to the reconstruction of crystal structures during thermal processing^22^.

However, Fig. 1(f) shows Raman bands, in which case samples 5 G and 6 G at 1346, 1597 and 2715 cm^−1^ correspond to the D, G, and 2D bands of graphene-based materials, respectively^20,23^. The former D band originates from the defects in sp^2^ lattice structure (amorphous nature), the latter G band corresponds to the first order scattering of sp^2^ (graphitic nature) domains while 2D bands originate from two phonon double resonance processes and are related to the band structure of graphene layers^24^. Raman spectrum of NG shows a shoulder to G peak, known as D’ which generally appears when there is lattice defect or doping of another atom and that confirm N-doping^20^. The 2D band for NG samples is hardly detectable in the spectra due to significant structural defects induced by thermal synthesis and intercalation of N atoms in addition to further increase in layers leads to a significant decrease of the relative intensity of the lower frequency 2D peaks^20^. As shown in Fig. 1(f, inset), the I_D_/I_G_ ratio of graphene blank samples is slightly lower than NG samples, demonstrating that N-doping increases the defects of graphene lattice and structural distortion. In addition from Supplementary Table S3, the extent of defects is decreased along with the decreasing of urea, indicating that the amount of defective edge of graphene is closely associated with the nitrogen content.

**Figure 2:**
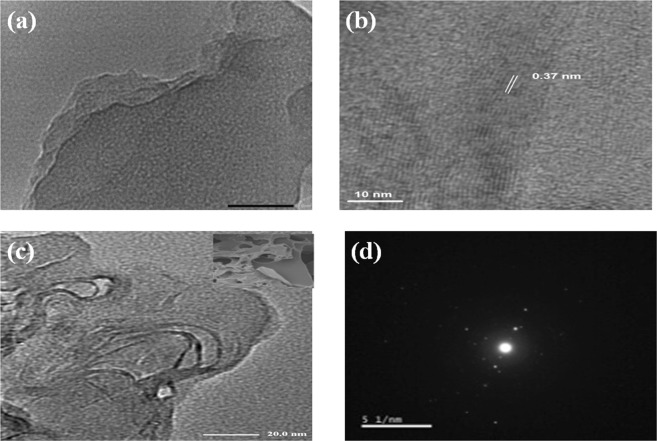
(**a**) TEM image and (**b**) HR-TEM image of sample 6 G, (**c**) corresponding TEM image with inset SEM image and (**d**) selected area diffraction pattern (SAED) of sample 4NG.

As shown in Fig. 2(a), TEM image for sample 6 G shows transparent large few thick layers and it is clear in high resolution TEM image (HR-TEM) in Fig. 2(b).While, SEM image for N doped samples (Fig. 2(c-inset)) shows structure like open 3D rich-pore network with corrugated microstructures and that confirmed by TEM image (Fig. 2(c)) which exhibited pores with different sizes in laminar morphology like silk veil waves with various wrinkles on the surface whereas wrinkles feature is due to presence of sp^3^ hybridization caused by structural distortions within the graphene network which induced by the thermal synthesis and embedded N atoms. Moreover, selected area electron diffraction pattern (Fig. 2(d)) shows dispersed bright spots overlay each other which confirm multilayered and polycrystalline structure^25^ of the obtained N doped graphene. It was noticed that the thermal decomposition of urea with PET released amount of gases which in turn has a great responsibility for the structure porosity. From morphology characterization, 3D porous structure not only provides excellent ions transfer pathway but also makes the surface area much more accessible and that confirmed by Nitrogen adsorption–desorption isotherm.

**Figure 3:**
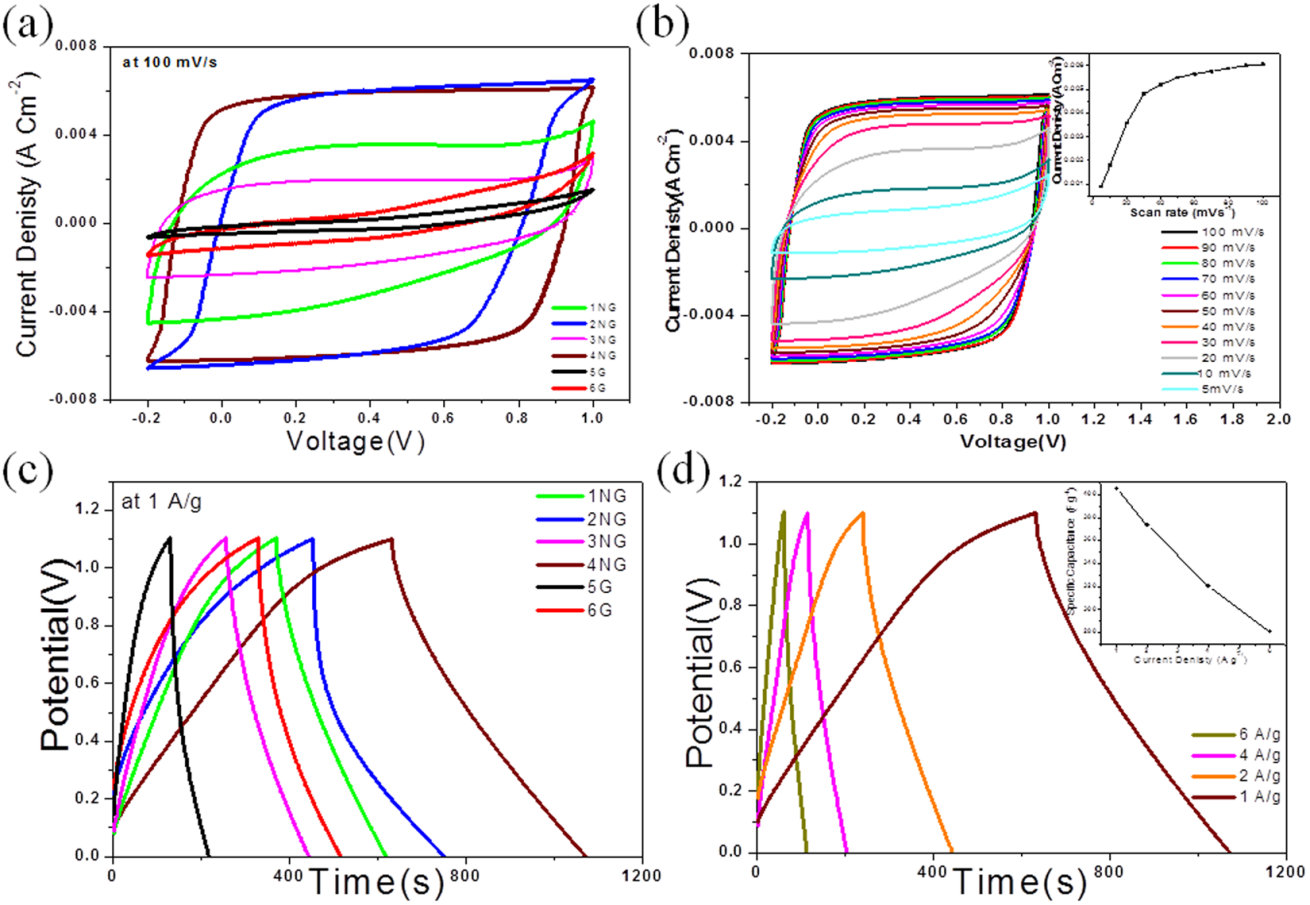
Electrochemical performance of the prepared samples. (**a**) CV curves at 100 mV s^−1^ in 6 M KOH and (**b**) CV curve for 4NG at different scan rates (inset shows the current densities are linearly grown with increasing scan rate), (**c**) Galvanostatic charge discharge curves at 1 A g^−1^ in 6 M KOH and (**d**) for 4NG at different scan rates(inset shows the effect of increasing the current density on the specific capacitance).

The unique 3D structure combines high surface area in addition to nitrogen doping content leading to the prominent capacitive and cycling performance of these supercapacitor electrode materials using two similar electrode cell. In Fig. 3(a) it can be noted that, the enclosed area of the cyclic voltammetry (CV) curves for the NG supercapacitors is much larger than that of the graphene supercapacitors, revealing that the performance of the capacitor is greatly improved by nitrogen doping and that might be attributed to the short ion transport path and the satisfactory electrical conductivity in NG electrodes^26^. Furthermore, it has been observed that graphene CV curves have distorted rectangle shapes, and that almost vanished in NG samples. This suggests a decrease in internal resistance and enhances specific capacitance after nitrogen doping^26,27^. The similar quasi-rectangle shape of CV curves in the voltage window range from −0.2 to 1 V were observed with excellent stability for all sample electrodes. It indicates that the capacitance mainly comes from the electrical double layer capacitance behavior in addition to fast charge propagation^11^. Obviously, sample 4NG exhibits a much higher current density and best rectangular shape than other NG samples which suggests that 4NG is the most promising material as supercapacitor electrode among all prepared samples. It can be revealed that the tailored effect of nitrogen doping can be used dramatically to increase the electrochemical properties of graphene. The CV curves of 4NG at various scan rates (5–100 mV S^−1^) were displayed in Fig. 3(b) and they appeared as quasi-rectangular shape with no distortion. In addition, the current densities are linearly grown with increasing the scan rates which may be attributed to the fast electronic transmission^27^.

To assess the capacitive property, a two-electrode (2E) cell was used to conduct the galvanostatic charge–discharge (GCD) tests. As shown in Fig. 3(c) the GCD curves of all samples exhibit linear and symmetrical shapes at a current density of 1 A g^−1^ in 6 M KOH, and no distinct IR drops appear in any curves, indicating excellent electrochemical reversibility, good rate capability and outstanding carrier transport within the electrodes for the electrochemical double layer capacitors (EDLCs). Furthermore, the capacitance of the 4NG electrode is four times higher than that of graphene electrode. The detailed GCD curves of the 4NG sample at various current densities are shown in Fig. 3(d). The specific capacitances can be calculated from Equation (1) according to the discharge curves and they are about 405, 373, 320, 281 F g^−1^ in 6 M KOH at current densities of 1, 2, 4, and 6 A g^−1^, respectively. However, the unchanged curves with linear and symmetrical shapes at various current densities are observed from this test. As a result it can be revealed that, the NG possesses considerable capacitive performance in 6 M KOH and that ascribed to the combination of microporous in the NG sheet and the mesoporous between the NG sheet in 3D structure with high active area which facilitates ion transport, thus contributing synergistically to the high rate performance.

The energy density (E) and power density (P) are calculated according to Equations (2) and (3) from the special capacitance test in a voltage window of 1.1 V with current densities of 1–6 A g^−1^. The 4NG electrode with the point power density of 558.5 Wkg^−1^ displays maximum energy density of 68.1 Wh kg^−1^ at a current density of 1 A g^−1^, which is over three times higher than that of 6 G (29.6 Wh Kg^−1^). The results illustrated in Supplementary Table S3, revealed that the NG can be combined with high power density and high energy density as a supercapacitor electrode material. The NG electrode shows outstanding specific capacitance, high rate capability and excellent stability.

**Figure 4:**
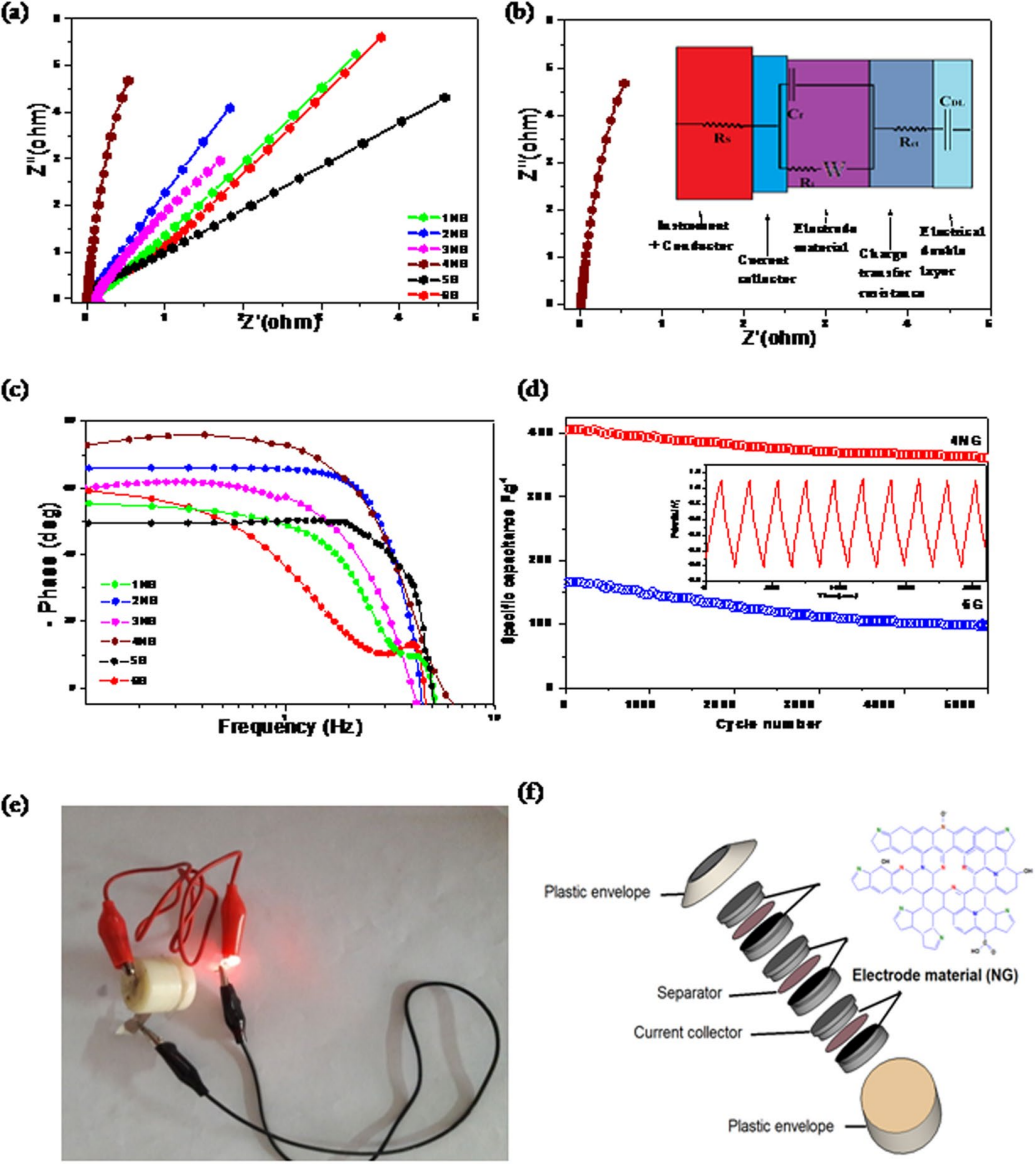
(**a**) Nyquist curves for all prepared samples as electrodes in 6 M KOH, (**b**) Nyquist curve of 4NG sample (inset shows its equivalent circuit), (**c**) Bode plots of all prepared samples supercapacitor devises, (**d**) The cycling stability of sample 4NG supercapacitor and sample 6 G, during 5000 charge/discharge cycles, measured at a current density 4 A g^−1^ within the potential range from 0 to 1.1 V, with the first 10 charge/discharge cycles for sample 4NG, (**e**) Photograph and (**f**) Schematic illustration of a stacking three-unit cell used to power a red LED.

The supercapacitive properties of all prepared samples as electrodes in 6 M KOH were studied through Electrochemical Impedance Spectroscopy (EIS). Nyquist plots as shown in Fig. 4(a) are used to elucidate the frequency dependent behavior of the prepared electrodes. It could be seen that the electrode of sample 4NG shows more vertical line than other electrodes, which suggesting that more capacitive character and electrode stability^4^ than other prepared samples which exhibit straight lines at the low frequency region and a small depressed semicircle in the high-frequency region. Moreover, more vertical line was observed for N doped electrodes than undoped electrodes, which suggested high electrochemical activity of N doped graphene towards supercapacitors. However, the obtained semi-circle represents the charge transfer impedance at the interface of the current collector/electrode material while, the vertical line represents ion diffusion in the structure of electrode materials and the slope of this line is related to the speed of EDL formation^28^.

As shown in the inset of Fig. 4(b), EIS results for prepared 4NG electrode were further analyzed using the equivalent circuit which shows the best fitted impedance data. From the circuit diagram, R_s_ corresponds to the resistance of the electrolyte is 0.003 Ω, R_ct_ is the charge transfer resistance (0.005 Ω), C_dl_ is the double layer capacitance (4-E6 Fg^−1^) and W represents the diffusion Warburg impedance (0.9) which is very low comparable to other prepared electrodes as illustrated in Supplementary Information Table S4.Bode phase angle plots as shown in Fig. 4(c) represent the impedance phase angle of different prepared electrodes. 4NG electrode is observed to be close to −74° at low frequency (1.3 Hz), which indicates the high capacitance performance^29^ and the relaxation time for the 4NG electrode was found to be (335 ms). Therefore, it is confident to say that, 4NG has got faster frequency response capability, with shorter ionic transport paths that in turn reduced internal resistance and increase the electrical conductivity^30^.

The cycling stability, which is a critical property of supercapacitors for their practical applications, was measured at a current density of 4 A g^−1^ using the galvano static charge/discharge technique. The capacitance retentions of 4NG were 87.7% of their initial capacitances, retained after 5000 charge/discharge cycles (Fig. 4(d)). This value was comparable to sample 6 G which shows cycling stability at about 59.8%.The obvious capacitance decay in the initial cycles could be ascribed to the removal of a few oxygen-containing groups which are more on graphene sheets than on NGs^1^.

The most important factors for evaluating power applications of electrochemical supercapacitors are energy and power densities. As shown in Supplementary Table S3, the energy densities of the NGs are remarkable, 4NG showing the highest value of 68.1 W h kg^−1^ at a current density of 1 A g^−1^. This value is higher than those reported earlier for other porous structured NG supercapacitors, as shown in Table 1, while the power densities are in the range of 554–559 W kg^−1^. Furthermore, in order to test its feasibility for practical applications as supercapacitor; three fabricated devices were connected in series as shown in Fig. 4(f) to power red light-emitting diodes. After charging at 6 A g^−1^ for 60 s, the device could light the LED for over 5 min, which can be seen in Fig. 4(e). This result obviously demonstrates the potential of the fabricated supercapacitor device in energy storage.

**Table 1:** Electrochemical performance of various supercapacitors fabricated based on nitrogen doped graphene materials.

Material	Electrolyte	Test condition A g^−1^	Specific capacitance F g^−1^	Energy density W h kg^−1^	Power density W kg^−1^	ref.
Nitrogen-doped graphene with bubble-like textures	2 M KOH	1	481	42.8	500	^12^
Porous nitrogen doped graphene film (PNGF)	6 M KOH	2	250	6.43	150	^24^
Porous nitrogen-doped graphene (PNG)	[BMIM]PF6	1	310	163.8	600	^30^
Mn_3_O_4_ nanodots loaded on nitrogen-doped graphene sheets	EMIMBF4	0.5	28.5	63.3	—	^33^
3D hierarchical porous nitrogen-doped carbon frameworks *in situ* armored NiO nanograins (NCF/NiO)	2 M KOH	1	113	40.18	800	^34^
highly porous nitrogen-sulfur co-doped graphene nanoribbons (NS-GNRs)	1 M Na_2_SO_4_	0.5	442	23.85	448	^35^
3D-Porous nitrogen doped graphene	6 M KOH	1	405	68.1	558.5	This work^36^

## Conclusion

This work presents one simple step and industrial scalable green synthesis for NG with 3D porous architecture. The morphology structure, the specific surface area and the surface functionalities of the synthesized NG can be tailored by controlling the synthetic conditions, including urea doses and thermal decomposition temperature. On the basis of the previous results, it can be concluded that the nitrogen doped and unique 3D porous architecture, which affords fluent ion transport through open porous structure with a large surface area and high electrical conductivity are a key factor in maintaining the high specific capacitance, rate performance and stability. Eventually, the easy synthesis strategy with great unique functional structures can open up enormous opportunities to design and develop high performance supercapacitors as well as other energy storage devices.

## Experimental

### Preparation of nitrogen doped graphene (NG)

The raw PET waste was mixed with urea at different ratios 1:1, 1:1.5 and 1:2, and then introduced into an enclosed 50 mL stainless steel autoclave reactor (SS316). The closed stainless steel reactor was placed inside the center of an electric furnace and the experiments were repeated at two different temperatures 600 °C and 800 °C with a rate of 10 °C min^−1^ and maintained at these temperatures for 5 h (The experimental conditions are summarized in Supplementary Table S1). After that the system was left to cool overnight. The resulted dark products were collected and crushed. During the decomposition process, the PET was thermally reduced and N atoms were doped into the graphitic lattice, producing NG. In order to compare the capacitance activity of NG, we also prepared graphene (G) and NG samples with different urea to PET ratio.

### Physico chemical characterization

The thermal properties of raw PET, urea and a mixture of PET and urea with 1:1 wt. ratio were investigated by TGA using a thermogravimetric analyzer (Shimadzu TGA-50) from 20 °C to 800 °C at a rate of 10 °C min^−1^ under nitrogen gas flow. The composition of the prepared materials was studied using Fourier transform infrared (FTIR) (Bruker Corporation, Ettlingen, Germany) and X-ray photoelectron spectroscopy (XPS) Phi 5300 ESCA system (Perkin-Elmer, U.S.A) with the Mg (Kα) radiation (X-ray energy 1253.6 eV). The XPS samples were prepared by dropping 100 μL of NG aqueous dispersions (1.0 mg/mL) onto silicon substrates followed by air drying. Elemental analysis of the NG samples was measured using vario-Micro CHN Elemental analyzer (Germany).

Nitrogen (N_2_) adsorption-desorption isotherms were recorded with Shimadzu, Micromeritics ASAP 2010 Instrument) cooled by liquid N_2_. Prior to recording sorption isotherm, the sample was degassed at 120 °C under vacuum for 12 h.

The morphology of the prepared samples was explored using scanning electron microscopy (SEM), JEOL (JSM 6360 LA, Japan) instrument with an operating voltage of 15 kV, and transmission electron microscopy (TEM), JEOL (JEM-2100 plus, Japan) under an accelerating voltage of 200 kV.

Raman spectroscopy (SENTERRA, Bruker) with a 514.5 nm excitation wavelength and X-ray diffraction (XRD, Schimadzu-7000, using a CuKα radiation operating at 30 kV and 30 mA and scanning rate of 4° min^−1^ were employed to analyze the crystal structure of prepared materials.

### Electrochemical measurement

To prepare the working electrode, the prepared active materials (90 wt %), carbon black (5 wt %), polyvinylidinefloride (5 wt %), and 1-methyl 2-pyrrolidinone as a solvent were mixed forming slurry. The slurry was then coated onto nickel sheet (1 cm^2^) and dried at 60 °C overnight. The mass of active materials in the active surface was around 1–1.2 mg cm^−2^.

The electrochemical measurements were performed in a two-similar electrode cell using 6 M potassium hydroxide (KOH) electrolyte solutions, nickel sheet as current collector and Whatman fiber glass filter paper (GF/D) as a separator. The electrochemical characterization was performed at room temperature using a computer-controlled Potentiostat (Metrohm Autolab, model: 87070) involving cyclic voltammetry (CV), galvanostatic charge/discharge (GCD), and electrochemical impedance spectroscopy (EIS). The CV studies were performed between − 0.2 to 1 V at increasing sweep rates from 5 mV S^−1^ to 100 mV S^−1^. The charge–discharge measurements were performed at different current densities (from 0.5 to 6 Ag^−1^). Cyclic stability tests were conducted at a current density of 4 A g^−1^. The specific gravimetric capacitance of the supercapacitor was determined from the galvanostatic cycles by means of the formula:

1 $${\rm{C}}=\frac{{\rm{I}}\times {\rm{\Delta }}{\rm{t}}}{{\rm{m}}\times {\rm{\Delta }}{\rm{V}}}$$

where I, ∆t, ∆V, and m are the discharge current (A), discharge time (s), discharge voltage (V), and the mass of the active material (g), respectively^31^.

Energy density (E) and power density (P) were calculated using the formulas,

2 $${\rm{E}}=\frac{{\rm{C}}\times {{\rm{\Delta }}{\rm{V}}}^{{\rm{2}}}}{2\times {\rm{3.6}}}$$

3 $${\rm{P}}=\frac{{\rm{E}}\times {\rm{3600}}}{{\rm{\Delta }}{\rm{t}}}$$

where C is the specific capacitance, V is the operational potential window and t is the discharge time^31,32^. Electrochemical Impedance Spectroscopy (EIS) tests were recorded in a frequency range from 0.001 to 100 kHz with a sinusoidal signal of 5 mV.

## Supplementary information

Supplementary Information
