# Supplementary material for: Development of High-Performance Supercapacitor based on a Novel Controllable Green Synthesis for 3D Nitrogen Doped Graphene
Source: Sci Rep. 2019 Feb 4;9:1129. doi: 10.1038/s41598-018-37369-x (PMC6362120; doi:10.1038/s41598-018-37369-x)
Supplement: Supplementary file 1 — Supplementary Information [file 41598_2018_37369_MOESM1_ESM.doc]

**Supplementary Information**

**Development of High-Performance Supercapacitor based on a Novel Controllable Green Synthesis for 3D Nitrogen Doped Graphene**

Noha A. Elessawy ^a*^**,** J. El Nady ^b^**,** W. Wazeer ^b^**,** A. B. Kashyout ^b^

^a^ Advanced Technology and New Materials Research Institute, City of Scientific Research and Technological Applications (SRTA-City), New Borg El-Arab City, P.O. Box 21934 , Alexandria, Egypt.

^b^ Electronic Materials Department, Advanced Technology and New Materials Research Institute, City of Scientific Research and Technological Applications (SRTA-City), New Borg El-Arab City, P.O. Box 21934, Alexandria, Egypt

* Noha A. Elessawy (nony_essawy@yahoo.com)

**Experimental**

**Preparation of graphene and nitrogen doped graphene (NG) from PET waste bottles**

In this research, carbon nanostructure materials have been fabricated by fragmentation or “cutting” of PET

bottle waste (top-down approach). The PET waste was crushed and sieved to obtain desired size fractions (1–3 mm) using a conventional sieve-shaker. Then mixed with urea in different ratio 1:1, 1:1.5 and 1:2, the experimental conditions are summarized in Supplementary Table S1 .The closed stainless steel reactor was placed inside the center of an electric furnace and the experiments were repeated at two different temperatures 600 °C and 800 °C with a rate of 10 °C min^−1^ and maintained at these temperatures for 5 h. After that the system was left to cool overnight. The resulted dark products were collected and crushed. During the decomposition process, the PET was thermally reduced and N atoms were doped into the graphitic lattice, producing NG. In order to compare the capacitance activity of NG, we also prepared graphene (G) and NG samples with different urea to PET ratio.

**Table S1:** Samples and their operating conditions used during the experimental tests

| **Sample code** | **PET : Urea Ratio** | **Temperature** | **Time** |
| --- | --- | --- | --- |
| 1NG | 1:2 | 600 ºC | 5 hrs |
| 2NG | 1:1.5 | 600 ºC | 5 hrs |
| 3NG | 1:1 | 600 ºC | 5 hrs |
| 4NG | 1:2 | 800 ºC | 5 hrs |
| 5G | 1:0 | 600 ºC | 5 hrs |
| 6G | 1:0 | 800 ºC | 5 hrs |

**Supporting Experimental Results**

*Thermal gravimetric analysis (TGA) for prepared samples*

Thermogravimetric analysis (TGA) is used to investigate the behaviors of PET and urea in the process of thermal decomposition. Supplementary Fig. S1 presents the homologous TG curves of urea, PET/urea (a mixture of PET and urea with 1:1 weight ratio) and PET. The curve of urea indicates three stages of mass loss. In the first stage which is related to urea decomposition to render biuret and volatile products whereas a mass loss about 79 % and occurs between 150 and 240 °C. The second stage to 300 °C involves a distinct mass loss of 20 %, which corresponds to the generation of cyanuric acid and volatile materials. Moreover, small amounts of ammelide, ammeline and melamine are formed. These small amounts decompose during the last step up to 400 °C . Urea totally decomposes into volatile products with a total weight loss close to 600 °C. While, for PET The degradation started at 360 °C and the temperature at which the maximum degradation rate was achieved at 480°C .The curve exhibits one major mass loss which was attributed to thermal decomposition and the residue at 800 °C was 0.9 wt %, this is obviously associated with the segment corresponding to the carboxylic acid and the benzene ring appearing in the chemical unit of PET ^[1S]^. In contrast to PET and urea curves, the curve of PET/urea is coalescent of three curves. A mass loss of 60% takes place in the first step at temperature range of 150–240 °C, which is in good agreement with urea decomposition to biuret. This process followed by two steps ended at 480 °C similar to PET curve. During thermal decomposition process, the out-gassing as CO, CO_2_, NO_2_, and H_2_O molecules could introduce force to expand the graphene layers and help the formation of porous frameworks ^[2S,3S]^ and that confirmed by SEM and TEM images. Meanwhile, the nitrogen atoms embedded in carbon lattice resulted in the formation of NG frameworks.

**Figure S1:** TG curves of urea, PET/urea (a mixture of PET and urea with 1:1 wt. ratio) and PET

(a)

(b)

(c)

**Figure S2:**(a) High resolution C1s spectra of the synthesized 4NG sample and High resolution N1s XPS spectra with N1 (pyridinic-N), N2 (pyrrolic-N), N3 (graphitic-N) and N4(oxidized nitrogen) of the N-doped graphene (b) sample1NG, (c) sample 4NG

**Figure S3:** XRD spectra for all samples.

**Figure S4:** Pore size distribution of 3D N doped graphene samples calculated using DFT method

**Table S2:** XPS and elemental analysis data of graphene and N-doped graphene samples.

| **Sample** | **C**_XPS_  **wt.%** | **C** _Elemental_  **wt.%** | **N**_XPS_  **wt.%** | **N** _Elemental_ **wt.%** | **O**_XPS_  **wt.%** | **O** _Elemental_  **wt.%** |
| --- | --- | --- | --- | --- | --- | --- |
| **1NG** | 77.13 | 74.95 | 9.41 | 11.43 | 13.46 | 11.37 |
| **2NG** | 78.391 | 75.65 | 7.042 | 9.6 | 14.567 | 12.58 |
| **3NG** | 91.87 | 89.94 | 0.2 | 2.03 | 7.93 | 6.74 |
| **4NG** | 83.273 | 80.64 | 2.819 | 6.52 | 13.908 | 11.34 |
| **5G** | 93.85 | 90.49 | --- | --- | 6.15 | 6.5 |
| **6G** | 96.07 | 92.64 | --- | --- | 3.93 | 3.95 |

**Table S3:** Different properties of prepared graphene and NG samples

| **Sample** | **wt. ratio**  **of**  **PET : urea** | **Experiment**  **Temperature**  **ºC** | **SBET**  **(m^2^g^-1^)** | **N_XPS_**  **(at.%)** | **N1s** | | | | **I_D_/I_G_** | **Energy density**  **(Wh/ Kg)** | **Power density**  **(W/kg)** | **Specific**  **capacitance (F/g) at (1A/g)** |
| --- | --- | --- | --- | --- | --- | --- | --- | --- | --- | --- | --- | --- |
|  |  |  |  |  | **N _Pyridinic_**  **(at. %)** | **N _Pyrrolic_ (at. %)** | **N _Graphitic_**  **(at. %)** | **N _oxidized nitrogen_**  **(at. %)** |  |  |  |  |
| 1NG | 1:2 | 600 | 118 | 8.966 | 3.228 | 2.78 | 2.241 | 0.717 | 0.97 | 38.8 | 554 | 231 |
| 2NG | 1:1.5 | 600 | 336.8 | 6.332 | 2.279 | 1.821 | 1.489 | 0.742 | 0.89 | 46.6 | 557 | 277 |
| 3NG | 1:1 | 600 | 121 | 0.137 | 0.063 | 0.042 | 0.032 | --- | 0.83 | 29.5 | 558.9 | 175 |
| 4NG | 1:2 | 800 | 702 | 2.514 | 0.733 | 0.770 | 0.881 | 0.13 | 1.04 | 68.1 | 558.5 | 405 |
| 5G | PET only | 600 | 108 | --- | --- | --- | --- | --- | 0.93 | 13.95 | 558 | 83 |
| 6G | PET only | 800 | 418 | --- | --- | --- | --- | --- | 1.04 | 29.6 | 558 | 176 |

**Table S4:** Equivalent circuit parameters of all prepared electrodes and these values are calculated by Program (Nova 1.11) by fitting impedance data on the designed equivalent circuit

| sample | ^a^ R_S_ (Ω) | ^b^ R_i_(Ω) | ^c^ R_ct_(Ω) | ^d^ W | ^e^ C_f_ (Fg^-1^) | ^f^ C_DL_(Fg^-1^) |
| --- | --- | --- | --- | --- | --- | --- |
| 1NG | 0.083 | 4.7 | 0.081 | 1.11 | 45-E6 | 0.01 |
| 2NG | 0.051 | 3.26 | 0.058 | 6.62 | 11-E6 | 0.003 |
| 3NG | 0.076 | 0.11 | 0.076 | 3.19 | 29-E6 | 9 -E6 |
| 4NG | 0.003 | 0.11 | 0.005 | 0.9 | 4-E6 | 4-E6 |
| 5G | 0.062 | 0.47 | 0.062 | 2.24 | 4.34-E4 | 4.87-E4 |
| 6G | 0.27 | 2.39 | 0.24 | 1.31 | 3.09-E4 | 0.012 |

^a^ R_S_ equivalent series resistance

^b^ R_i_ carbon – current collector interface resistance

^c^R_ct_ Resistance of charge transfer from double layer to carbon

^d^W Warburg impedance element

^e^C_f_ faradaic capacitance of carbon – collector interface

^f^C_DL_ Double layer capacitance

**References**

| [1S] W. Brack, B. Heine, F. Birkhold, M. Kruse, G. Schoch, S. Tischer, O. Deutschmann, *Chem. Eng. Sci.***2104,** *106*,1. |
| --- |
| [2S] L. Peng, Z. Xu, Z. Liu, Y. Guo, P.Li , C. Gao, *Adv. Mater.***2017,** *29,* 1700589. |
| [3S] N. Xiao, H. Tan, J. Zhu, L. Tan, X. Rui, X. Dong, Q. Yan, ACS *Appl. Mater. Interfaces* **2013**  *5* , 9656. |
